# Successful aortic arch cannulation and perfusion of a heart donated after circulatory death: A case report

**DOI:** 10.1177/02676591231200986

**Published:** 2023-09-05

**Authors:** Mats T. Vervoorn, Paul van Kaam, Mostafa M. Mokhles, Niels P. van der Kaaij, Monica Gianoli

**Affiliations:** 1 Department of Cardiothoracic Surgery, Division of Heart and Lungs, 8124 University Medical Center Utrecht, Utrecht, Netherlands; 2 Heartbeat Dutch Perfusion Services, Kerkstraat, Netherlands

**Keywords:** heart transplantation, ex situ heart perfusion, transmedics, donation after circulatory death, perfusion, congenital heart disease, heart failure

## Abstract

**Introduction:**

We describe successful aortic arch cannulation and perfusion of a heart donated after circulatory death using the Transmedics Organ Care System™.

**Case report:**

A 47-year old man developed advanced heart failure symptoms after prior mustard operation for transposition of the great arteries. He matched a DCD-donor and required an elongated aorta for implantation due to his altered anatomy. The donor heart was retrieved and successfully perfused via aortic arch cannulation for 4.5 h with satisfactory perfusion parameters.

**Disussion:**

Although Transmedics advises against aortic arch cannulation due to concerns regarding malperfusion, satisfactory and safe perfusion can be achieved by careful positioning of the heart. Awareness and attention to the occurrence of malperfusion is mandatory, especially during transport, to achieve satisfactory outcome.

**Conclusion:**

Aortic arch cannulation is feasible without compromising quality of perfusion. This is relevant for patient that require an elongated aorta after surgically corrected congenital heart disease.

## Introduction

After the successful implementation of cardiac donation-after-circulatory-death (DCD) in the United Kingdom^1^ and Australia,^2^ the Netherlands started a nationwide program for DCD heart donation and transplantation (HTx) in 2021 using the direct procurement and perfusion technique. The Transmedics Organ Care System™ ((OCS); Andover, MA, USA) is used as a platform for normothermic oxygenated blood perfusion of the explanted heart that allows for reconditioning and quality assessment of the graft after circulatory death, with short- and intermediate term outcomes comparable to standard-care HTx using hearts preserved with cold storage from brain death donors.^3,4^

This case report describes the successful recovery of a DCD heart for a recipient with altered anatomy due to a prior Mustard procedure for transposition of the great arteries (TGA) that necessitated a modified dissection of the heart and perfusion on the OCS™. Specifically, this report demonstrates the feasibility of aortic arch cannulation for satisfactory perfusion on the OCS™.

## Case report

**Figure 1.**
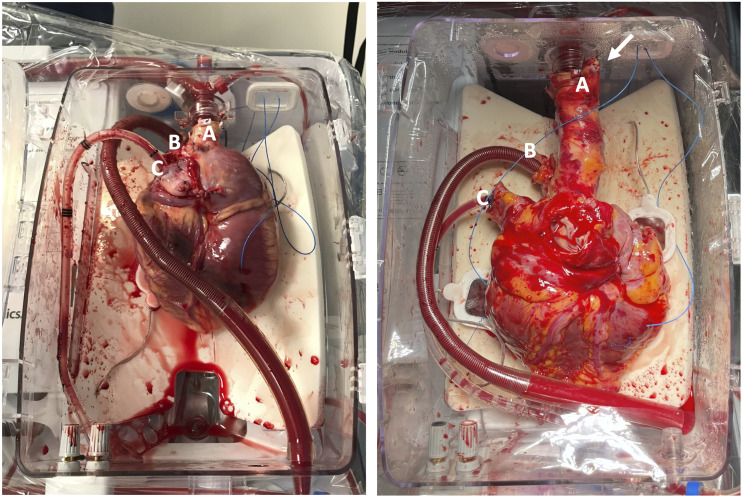
A heart procured with a short aorta (left) for reference. The procured donor heart with elongated aorta mounted on the Transmedics OCS™ prior to cooling for implantation into the recipient (right). The pulmonary artery catheter was disconnected for dismounting (right). (a) normal aorta (left), elongated aorta (right). (b) pulmonary artery catheter. (c) left vent inserted through the left atrial appendage. The arrow (right) points to the remnants of the ligated brachiocephalic trunk. The orientation of the heart is in accordance with the standard of practice as recommended by Transmedics, with the front side of the heart facing the machine.

A 47-year old man developed advanced heart failure symptoms after prior Mustard operation for TGA, which was conducted before his first birthday. At the age of 35, he started developing symptoms and was diagnosed with congestive heart failure, which progressed over the years. After the initial diagnosis, he was admitted to the hospital multiple times for congestion relief and treatment of recurrent supraventricular arrhythmias. He was enlisted on the waiting list for HTx at the age of 46 in NYHA class III, with a severely dilated and dysfunctional systemic right ventricle as assessed by echocardiography, systolic right ventricular pressures up to 105 mmHg and a cardiac index of 1.75 L/min/m^2^. His past medical history at that point reported epilepsy, there was no renal dysfunction. After 318 days on the waiting list, he matched a 58-year old male donor, who suffered a massive ischemic cerebrovascular event, but did not meet formal brain death criteria. Due to the altered anatomy of the recipient, an elongated aorta was requested for implantation. Bicaval excision of the donor heart was commenced after 22 min of functional warm ischemic time, defined as the time between systolic blood pressure <90 mmHg for a minute and administration of cardioplegia (St. Thomas II). Normothermic regional perfusion of the donor was not conducted. The arch vessels were identified and cut separately, the aortic arch was transected proximal to the left subclavian artery. The aortic arch was cannulated during backtable preparation using a medium sized aortic cannula, ligating the left carotid artery in the process. The brachiocephalic trunk was closed using pledgeted prolene 4-0 sutures. After 37 min of cold ischemia, the heart was mounted on the OCS™ for reperfusion. The elongated aorta necessitated lower positioning of the heart and manual repositioning of the electrode pads. A slight clockwise rotation was required to avoid aortic torsion (Figure 1). After careful positioning of the heart, reperfusion was established and was characterized by a low perfusion pressure of +/− 50 mmHg with a high coronary flow of 850 mL/min, with an initial lactate level of 7.3 mmol/L. A subsequent downward trend in lactate levels was noted and the heart was accepted after 60 min of perfusion, with a lactate level of 6.9 mmol/L. During transportation and the remainder of perfusion, perfusion pressure was increased to 80 mmHg with coronary flows ranging between 650 and 700 mL/min.

The heart was dismounted after 4.5 h of uneventful perfusion, with a final lactate level of 2.45 mmol/L. The anatomy of the recovered heart matched the requirements for HTx into the recipient and the donor heart was implanted successfully and could be reperfused in the recipient 1 h and 42 min after dismounting from the OCS™. Weaning from cardiopulmonary bypass was swift with inotropic support (dobutamine, milrinone, norepinephrine). The recipient was transferred to the ICU and weaned off the ventilator and inotropic support on the first postoperative day. Echocardiography after implantation showed adequate right and left ventricular function, the cardiac index was 2.8 L/min/m^2^. He was transferred to a medium care ward for further recovery on the third postoperative day and could be discharged from the hospital after 35 days. He remains free of complications after 1 year of follow-up, with no signs of rejection, graft dysfunction or allograft vasculopathy.

## Discussion

In this case report, we demonstrate feasibility of aortic arch cannulation for perfusion on the OCS™ for a recipient that required an elongated aorta due to prior Mustard procedure. Given the progressive nature and etiology of his heart failure, we opted to transplant the patient when he matched the 58-year old donor.

Although Transmedics^5^ advises against cannulation of the aortic arch to avoid malperfusion due to “ballooning” of the aorta, torsion and concerns over differential blood flow when the aorta is elongated, we have demonstrated that it is possible to establish satisfactory and safe perfusion by careful positioning and monitoring of the heart to avoid these complications. However, it is important to note that awareness and attention to the potential occurrence of malperfusion using this approach are mandatory, especially during transportation. Attention to the positioning of the heart after reperfusion, and close monitoring of perfusion parameters (i.e. coronary flow and aortic pressure) and lactate trends to enable timely intervention is therefore recommended. By sharing our experience, we support the feasibility of aortic arch cannulation and advocate the use of DCD hearts for HTx to recipients who require an elongated aorta due to surgically corrected congenital diseases.

## Appendix

### Abbreviations

DCD: Donation-after-circulatory-death
HTX: Heart transplantation
OCS: Organ Care System
TGA: Transposition of the great arteries

## References

[1] Messer S Cernic S Page A , et al. A 5-year single-center early experience of heart transplantation from donation after circulatory-determined death donors. J Heart Lung Transplant 2020; 39(12). 10.1016/j.healun.2020.10.001 33248525

[2] Chew HC Iyer A Connellan M , et al. Outcomes of Donation After Circulatory Death Heart Transplantation in Australia. J Am Coll Cardiol 2019 Apr; 73(12): 1447–1459. 30922476 10.1016/j.jacc.2018.12.067

[3] Joshi Y Scheuer S Chew H , et al. Heart Transplantation From DCD Donors in Australia: Lessons Learned From the First 74 Cases. Transplantation 2023 Feb 31; 107(2): 361–371. 36044329 10.1097/TP.0000000000004294

[4] Schroder JN Patel CB DeVore AD , et al. Transplantation Outcomes with Donor Hearts after Circulatory Death. N Engl J Med 2023 Jun 8; 388(23): 2121–2131. 37285526 10.1056/NEJMoa2212438

[5] Transmedics . TransMedics®. Organ care SystemTM OCS heart user guide, 2021, p. 153.

